# Anthocyanins from *Lycium ruthenicum* Murray Mitigate Cadmium-Induced Oxidative Stress and Testicular Toxicity by Activating the Keap1/Nrf2 Signaling Pathway

**DOI:** 10.3390/ph17030322

**Published:** 2024-03-01

**Authors:** Mingran Dong, Juan Lu, Hongwei Xue, Yang Lou, Shuyang Li, Tao Liu, Zimian Ding, Xi Chen

**Affiliations:** 1 State Key Laboratory for Quality Ensurance and Sustainable Use of Dao-di Herbs, Institute of Medicinal Plant Development, Chinese Academy of Medical Sciences, Peking Union Medical College, Beijing 100193, China; dongmingran1998@163.com (M.D.); jlu@implad.ac.cn (J.L.); 15297318065@163.com (H.X.); louyang0727@163.com (Y.L.); 15563999708@163.com (S.L.); 2 School of Pharmacy, University of Michigan, Ann Arbor, MI 48105, USA; taolius@umich.edu

**Keywords:** Cadmium, *Lycium ruthenicum* Murray, anthocyanins, oxidative stress

## Abstract

Cadmium (Cd) is a hazardous heavy metal environmental pollutant that has carcinogenic, teratogenic, and mutagenic properties. Excessive exposure to Cd can induce oxidative stress, which greatly harms the male reproductive system. Anthocyanins have remarkable antioxidative, anti-inflammatory, and anti-stress properties. In this study, we investigated the effects of anthocyanins and the underlying mechanisms through which anthocyanins mitigate Cd-induced reproductive damage. We isolated and purified *Lycium ruthenicum* Murray anthocyanin extract (LAE) and performed UHPLC-MS/MS to identify 30 different anthocyanins. We established an ICR mouse Cd injury model by administering 5 mg/kg/day CdCl_2_ for 28 consecutive days. LAE at 500 mg/kg/day effectively ameliorated testicular damage and preserved spermatogenesis. The mice in the LAE-treated group had elevated testosterone and inhibin B levels. Additionally, the treatment restored the activity of antioxidant enzymes, including T-SOD, CAT, and GR, and substantially increased the levels of the non-enzymatic antioxidant GSH. Research findings indicate that LAE can activate the SIRT1/Nrf2/Keap1 antioxidant pathway. This activation is achieved through the upregulation of both the SIRT1 gene and protein levels, leading to the deacetylation of Nrf2. Moreover, LAE reduces the expression of Keap1, alleviating its inhibitory effect on Nrf2. This, in turn, facilitates the uncoupling process, promoting the translocation of Nrf2 to the nucleus, where it governs downstream expression, including that of HO-1 and GPX1. LAE effectively mitigated toxicity to the reproductive system associated with exposure to the heavy metal Cd by alleviating oxidative stress in the testes.

## 1. Introduction

Cadmium (Cd) is a toxic heavy metal that is found worldwide and seriously threatens the environment [1]. It is generated primarily as a byproduct of industrial processes, such as electroplating, mining, zinc-Cd batteries, and smelting [2]. As Cd is found in the soil, water sources, atmosphere, and food, its exposure to humans is a growing concern [3]. Exposure to Cd can occur at workplaces via cigarette smoke or via rice containing Cd [4]. In the environment, Cd has a long half-life of 10–30 years and resists elimination by organisms, resulting in its bioaccumulation [5].

Cadmium (Cd) poses serious health risks to various organs, including the liver [6], kidneys [7], brain [8], heart [9], and gastrointestinal system [10], and it also inflicts severe damage to the reproductive system [11]. Mammalian testicles are highly sensitive to Cd toxicity due to active germ cell division and metabolism [12]. In one study, mice were administered different doses of Cd (0, 0.5, 1.0, or 2.0 mg/kg/day) for 28 days. The results showed that exposure to a high dose of Cd significantly decreased testosterone levels and the number of Leydig cells, increased the gap between seminiferous tubules, decreased the number of spermatozoa, led to a disordered arrangement of cells, and caused vacuolation. These changes indicated severe pathological structural damage to the testicles [13]. In another study, 10-week-old rats were administered CdCl_2_ for 28 days. The accumulation of Cd in the serum, testicles, and epididymis leads to a decrease in the weight of the rats, a decrease in the weight of their testicles and epididymis, and a reduction in sperm count and motility [14]. Cd can also induce testicular oxidative stress by reducing the levels of oxidases, such as total superoxide dismutase (T-SOD), catalase (CAT), and glutathione peroxidase (GSH-PX), while increasing malondialdehyde (MDA) levels [15].

Many studies have reported that the mechanisms underlying Cd-induced reproductive toxicity are complex [16,17,18,19]. The presence of Cd significantly increases the expression of blood vessel-related proteins, including CD31, NG2, αSMA, and caveolin-1, which stimulate abnormal proliferation of vascular endothelial cells and result in high blood pressure and vascular damage [20]. Cd can decrease the continuous expression of crucial integral membrane proteins, including occludin, N-cadherin, and claudin-11, thus compromising the integrity of the blood–testis barrier (BTB) [21].

However, the mechanism of Cd-induced testicular toxicity needs to be determined. Several studies have shown that the aforementioned pathways affect the production of reactive oxygen species (ROS), disrupt the oxidant/antioxidant balance, and lead to oxidative stress [22]. Excessive ROS in cells can significantly damage proteins and DNA, leading to cytotoxicity and ultimately cell death. The nuclear transcription factor erythroid-2-like factor (Nrf2)/Kelch-like-ECH-associated protein 1 (Keap1) signaling pathway plays a key role in regulating antioxidants; it is considered to be the essential endogenous antioxidant signaling pathway [23]. Under normal physiological conditions, Keap1 binds to Nrf2 in the Nrf2-EHC homology 2 region, which negatively regulates the activity of Nrf2 by facilitating its degradation through ubiquitin protease [24]. However, when cells are exposed to ROS or other oxidative stressors, Nrf2 dissociates from Keap1. Activated Nrf2 then translocates into the nucleus, where it forms a heterodimer with the Maf protein. This complex binds to the antioxidant response element (ARE) and triggers the transcription of downstream antioxidant genes [25]. This leads to the activation of downstream phase II metabolic enzymes, including glutathione S-transferase (GST) and quinone oxidoreductase-1 (NQO1), which enhance the activities of antioxidant enzymes, such as CAT, T-SOD, GSH-PX, heme oxygenase-1 (HO-1), and γ-glutamylcysteine synthetase (γ-GCS) [26]. This process increases the antioxidant capacity of cells, thus promoting cell survival. Silent mating type information regulation 2 homolog-1 (SIRT1) is a nicotinamide adenine dinucleotide-dependent histone deacetylase that is pivotal for cell cycle control, apoptosis, energy metabolism, and protection of mitochondrial function. It also plays a crucial role in the inflammatory response and resistance to oxidative stress. The SIRT1 signaling pathway interacts with the Nrf2 signaling pathway.

Since the 1955 outbreak of itai-itai disease in Japan caused by Cd pollution, researchers have been searching for drugs to neutralize heavy metal toxicity [27]. Traditional chelating agents such as meso-2,3-dimercaptosuccinic acid (DMSA), dimercaprol (BAL), and ethylenediaminetetraacetic acid disodium (EDTA-2Na) have shown limited effectiveness in treating chronic Cd poisoning. They may even worsen Cd accumulation in the kidneys [26,28,29,30,31]. Several researchers have investigated natural compounds, such as curcumin and melatonin, and elements, such as zinc and selenium [32,33,34,35]. Among these compounds, anthocyanins have garnered increased attention for their potent antioxidant properties and potential to mitigate Cd poisoning [36]. Anthocyanidins are water-soluble flavonoid pigments that are often glycosylated or acylated to form anthocyanins [37]. These plants are commonly found in colored fruits, vegetables, and medicinal plants such as mulberries, black beans, and bayberries [38,39]. *Lycium ruthenicum* Murray is the fruit of perennial shrubs in the genus *Lycium* (family Solanaceae). It is an essential economic plant in China that contains high levels of anthocyanins, proanthocyanidins, polysaccharides, amino acids, phenolic acids, and other compounds [40,41]. The anthocyanin core structures mainly consist of petunidin, cyanidin, delphinidin, and malvidin [42]. The antioxidant capacity of anthocyanins varies based on their type of glycosylation and acylation, the number and position of hydroxyl groups, and the presence of unpaired electrons in rings [43].

In this study, we investigated the protective effect of LAE against male reproductive toxicity induced by exposure to Cd. We assessed the capacity of these compounds to mitigate damage to sperm cells and alleviate oxidative stress, and we elucidated the mechanism underlying their protective effects.

## 2. Results

### 2.1. Total Anthocyanin Content

The total anthocyanin content in LAE was quantified by the pH differential method and was found to be 43.64 ± 9.28 Pt g/100 g DW. The anthocyanin content was greater than that recorded in other plant sources, including *Sambucus nigra* L. [44], strawberry [45], and *Hibiscus sabdariffa* L. [46]. These results indicated that *Lycium ruthenicum Murray* is a good source of anthocyanins and might be useful for further investigation.

### 2.2. Phytochemical Analysis of Anthocyanins

**Figure 1:**
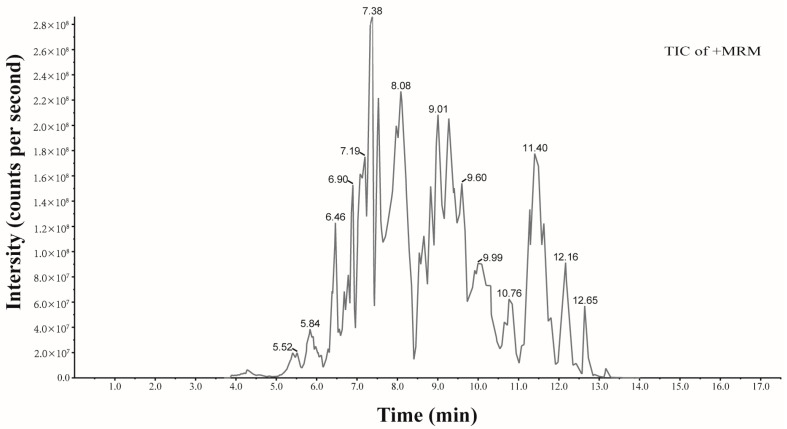
Total ion chromatogram of LAE in positive ion mode.

Using the UHPLC-MS/MS technique, we identified 30 known anthocyanin structures, including petunidin, cyanidin, delphinidin, malvidin, pelargonidin, and peonidin. Other flavonoid compounds, such as naringenin, were also detected. Additionally, phenolic compounds, mainly proanthocyanidins B1 and B2, were present. The structures, retention times (tR), and formulas of these compounds are presented in Table 1. The total ion chromatogram (TIC) is shown in Figure 1.

### 2.3. Therapeutic LAE Mitigates Cd-Induced Effects on Weight, Anogenital Distance, Testis, and Epididymal Organ Indices

**Figure 2:**
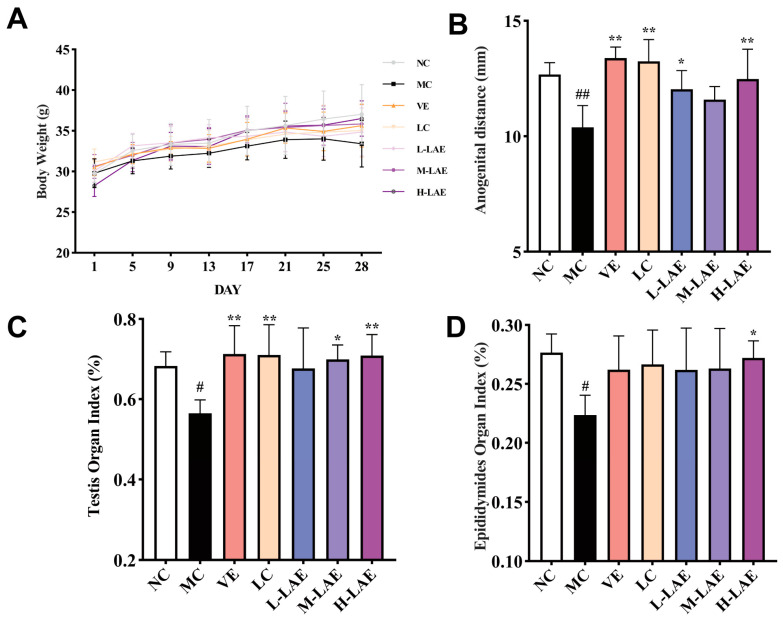
Changes in body weight, anogenital distance, the testis organ index, and the epididymis organ index were measured for each group. (**A**) body weight; (**B**) anogenital distance; (**C**) testis organ index; (**D**) epididymides organ index. All the data are presented as the means ± SDs (*n* = 6); * *p* < 0.05 and ** *p* < 0.01 compared to the model control group; # *p* < 0.05 and ## *p* < 0.01 compared to the normal control group. NC: normal control group (0.9% NaCl); MC: model control group (CdCl_2_, 5 mg/kg); VE: positive-drug group (vitamin E, 100 mg/kg, plus CdCl_2_, 5 mg/kg); LC: positive-drug group (L-carnitine, 500 mg/kg, plus CdCl_2_, 5 mg/kg); L-LAE, M-LAE, H-LAE: low-/middle-/high-dose treatment groups (LAE, 50/250/500 mg/kg, plus CdCl_2_, 5 mg/kg).

Throughout the experiment, the mice (6–8 weeks old) exhibited a steady increase in body weight, which is a characteristic of adolescence. However, beginning on day 21, the mice in the Cd-exposed model group exhibited delayed responses, dull fur, and a considerable decrease in body weight (Figure 2A). The MC group exhibited a significant reduction in anogenital distance compared to that of the NC group (*p* < 0.01; Figure 2B). The testis and epididymis indices decreased (*p* < 0.05; Figure 2C,D). The H-LAE group exhibited a significantly greater anogenital distance (*p* < 0.01), testis organ index (*p* < 0.01), and epididymis index than the MC group (*p* < 0.05).

### 2.4. Effects on Sperm Parameters

**Figure 3:**
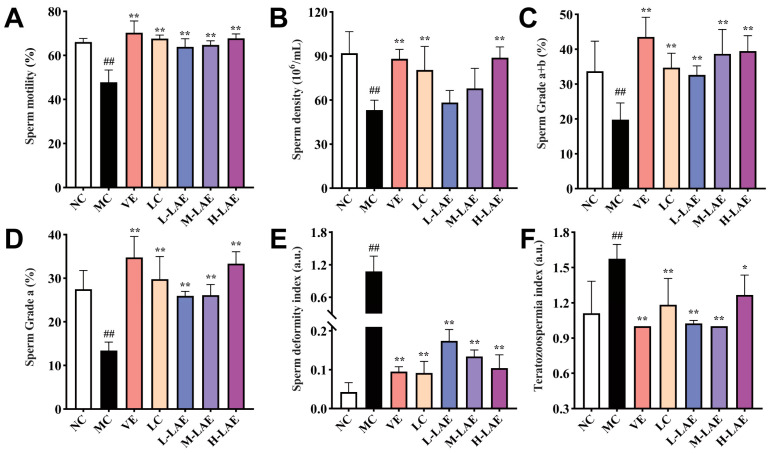
Changes in sperm parameters in each group. (**A**) sperm motility; (**B**) sperm density; (**C**) sperm grade “a + b”; (**D**) sperm grade “a”; (**E**) sperm deformity index; (**F**) teratozoospermia index. All the data are presented as the means ± SDs (*n* = 6); * *p* < 0.05 and ** *p* < 0.01 compared to the model control group; ## *p* < 0.01 compared to the normal control group. The grouping and abbreviations used are consistent with the descriptions provided earlier.

**Figure 4:**
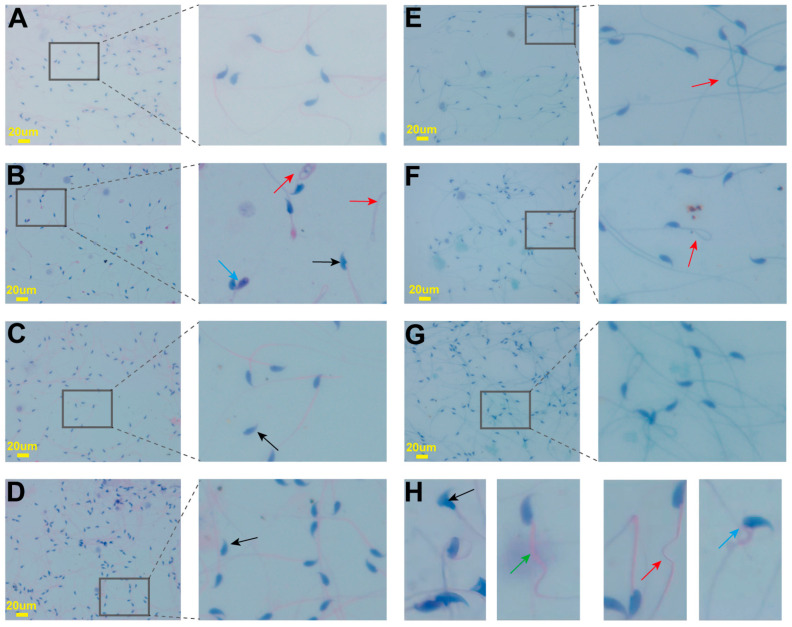
Evaluation of sperm morphology using the modified ultrafast Papanicolaou staining method. (**A**) NC group; (**B**) MC group; (**C**) VE group; (**D**) LC group; (**E**) L-LAE group; (**F**) M-LAE group; (**G**) H-LAE group. (**H**) Representation of abnormal sperm. The black arrows indicate head defects, the green arrows indicate midpiece defects, the red arrows indicate tail defects, and the blue arrows indicate excess cytoplasmic droplets in sperm. Permanent smears of sperm cells from all groups were observed under an optical microscope at 400× magnification (*n* = 6).

Following Cd exposure during adolescence, the mice exhibited a significant decrease in sperm motility (Figure 3A), sperm density (Figure 3B), sperm grade “a + b” (Figure 3C), and sperm grade “a” (Figure 3D). In the MC group, sperm density, motility, and vitality were considerably lower than those in the NC group (*p* < 0.01). In contrast, sperm density, motility, and the proportion of sperm showing rapid or slow/sluggish progressive motion were significantly greater in the LC-positive drug group and the H-LAE group than in the MC group (*p* < 0.01). Changes in sperm morphology for all groups are shown in Figure 4. The MC group presented a significantly greater number and a greater proportion of sperm cells with head defects, midpiece defects, tail defects, and excess cytoplasmic droplets. The sperm deformity index (Figure 3E) and teratozoospermia index (Figure 3F) were greater in the MC group than in the NC group (*p* < 0.01). We found that the L-LAE, M-LAE, and H-LAE groups had considerably lower proportions of abnormal sperm cells and fewer defects than the MC group.

### 2.5. Effects on Sex Hormones

**Figure 5:**
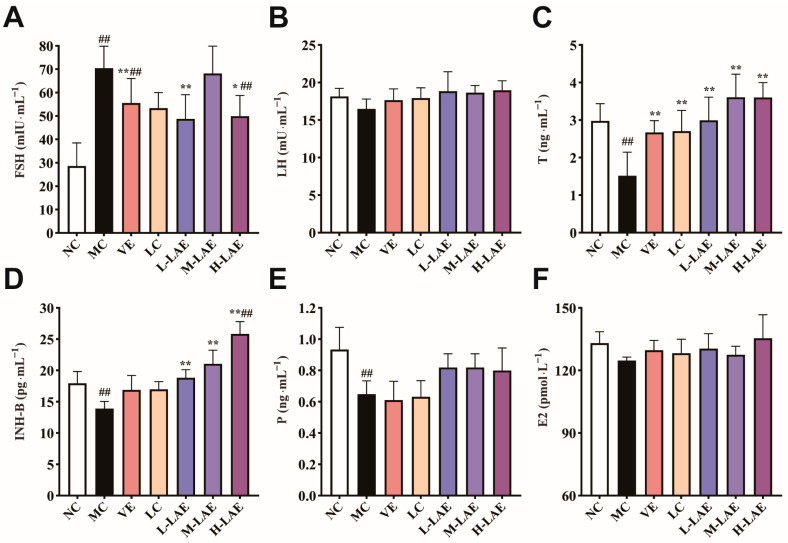
Changes in sex hormones in each group. (**A**) follicle-stimulating hormone (FSH); (**B**) luteinizing hormone (LH); (**C**) testosterone (T); (**D**) inhibin B (INH-B); (**E**) progesterone (P); (**F**) estradiol (E2). All the data are presented as the means ± SDs (*n* = 6); * *p* < 0.01 and ** *p* < 0.01 compared to the model control group; ## *p* < 0.01 compared to the normal control group. The grouping and abbreviations used are consistent with the descriptions provided earlier.

The serum concentration of FSH increased significantly in the MC group but decreased significantly following LAE intervention compared to that in the MC group (*p* < 0.05; Figure 5A). FSH acts on Sertoli cells, stimulating the production of INH-B, which, in turn, exerts negative feedback on the pituitary gland to regulate the secretion of FSH. We found that the MC group had significantly lower INH-B levels than the NC group, whereas H-LAE treatment effectively reversed these changes. The H-LAE group presented significantly greater INH-B levels than the MC and NC groups (*p* < 0.01; Figure 5D). Exposure to Cd significantly reduced the serum T level (*p* < 0.01; Figure 5C). Similarly, after administering VE, LC, L-LAE, M-LAE, or H-LAE, the T levels increased and were significantly greater than the T levels in the MC group (*p* < 0.01). LH can stimulate Leydig cells to synthesize and release T; however, the observed changes were not significant (Figure 5B). Circulating female hormone levels, such as E2 and P levels, can also regulate male hormone levels by modulating the hypothalamic–pituitary–gonadal axis (HPGA). However, the differences were not significant because their level of expression was low.

### 2.6. Histopathological Changes

**Figure 6:**
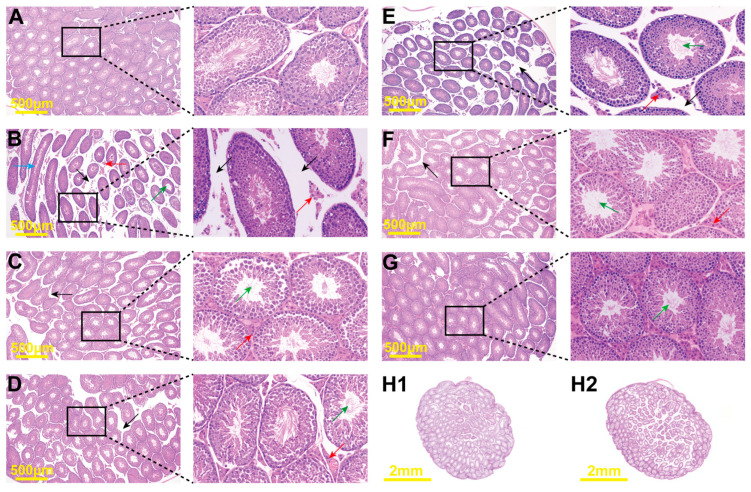

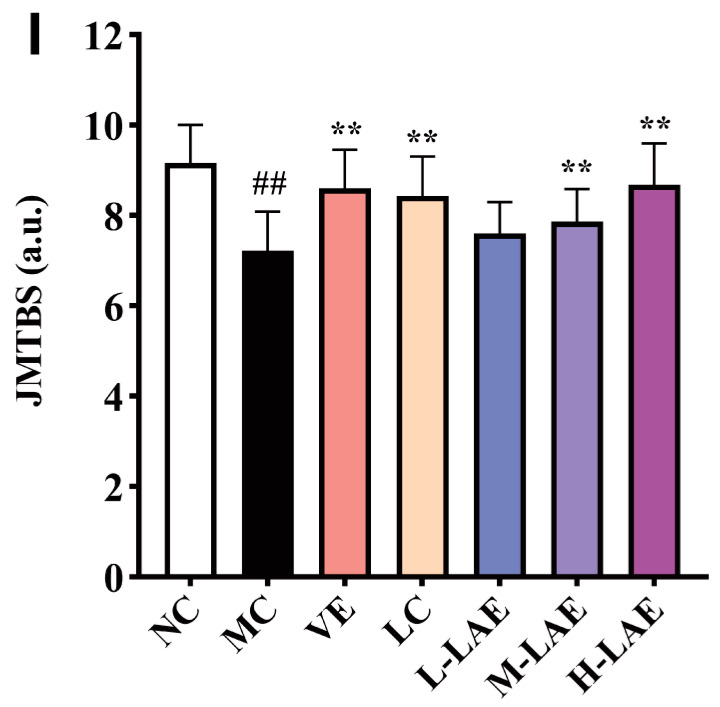
Histopathological sections and JMTBS scores. (**A**) NC group; (**B**) MC group; (**C**) VE group; (**D**) LC group; (**E**) L-LAE group; (**F**) M-LAE group; (**G**) H-LAE group; (**H1**) an image of testicle transection in the NC group; (**H2**) an image of a testicle transection in the MC group; (**I**) JMTBS scores. The black arrows indicate increased intertubular space, the green arrows indicate increased luminal space, the red arrows indicate thickening of the proliferative cell layers, and the blue arrows indicate localized dilatation of seminiferous tubules. All the data are presented as the means ± SDs (*n* = 6); ** *p* < 0.01 compared to the model control group; ## *p* < 0.01 compared to the normal control group.

The H&E-stained sections showed pathological changes in the cross-sections of the testicular tissues. The testicular tissues of the mice in the NC group exhibited intact testicular membranes, densely packed seminiferous tubules, and abundant interstitial cells (Figure 6H1). With sexual maturation, the lumina of the seminiferous tubules exhibited normal meiosis and multiple layers of spermatogenic cells, including spermatogonia, primary spermatocytes, secondary spermatocytes, and spermatids. The tubules produced a large number of mature sperm cells with flagella, and no abnormalities were observed (Figure 6A). In contrast, testicular tissues of the MC group showed significant atrophy or localized dilatation of seminiferous tubules (indicated by blue arrows). The number of interstitial cells was significantly reduced, and some proliferative cell layers had thickened (indicated by red arrows). This led to an increase in intertubular space (indicated by black arrows), fragmentation, and dispersion of the seminiferous tubules. Disruption of the multilayered epithelial structure of the seminiferous tubules resulted in a decrease in the number of epithelial cells, abnormal morphology of Sertoli cells, detachment of germ cells, luminal enlargement, and a significant decrease in mature sperm cells (indicated by green arrows). This caused severe pathological changes and sperm blockage (Figure 6B). Although the degree of testicular lesions improved slightly in the VE and LC groups, the size of the seminiferous tubule lumina still increased, and the interstitial cell layers became thicker. However, the LC group showed a significant increase in the number of mature sperm cells in the lumina (Figure 6C,D). After L-LAE intervention, the integrity of the spermatogenic epithelium was partially restored. After administering M-LAE and H-LAE, we found significant protective effects of LAE on testicular tissue structure. The seminiferous tubules were closely arranged, spermatogenic cell morphology was normal, and the number of interstitial cells was also normal. However, abnormally dilated seminiferous tubules were still present (Figure 6E–G).

Based on the histological observations, we assessed the condition of the testicular seminiferous tubules using Johnsen’s scoring method (Figure 6I). Due to a significant reduction in the number of late-stage sperm cells in the tubules after exposure to Cd, the scores decreased significantly (*p* < 0.01). In contrast, the VE, LC, M-LAE, and H-LAE groups exhibited significantly improved integrity of the spermatogenic epithelium, thus protecting the sperm production process. The scores of the above groups were significantly different from those of the MC group (*p* < 0.01).

### 2.7. Effects on Testicular Marker Enzymes

**Figure 7:**
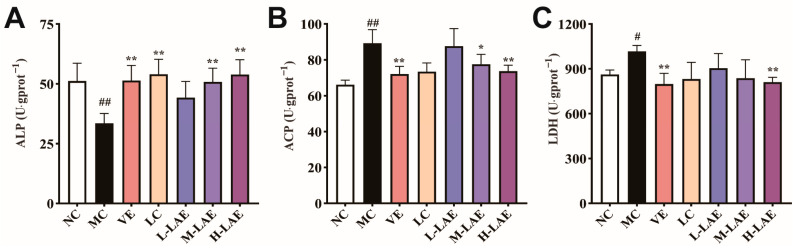
Changes in the ALP, ACP, and LDH levels. (**A**) ALP; (**B**) ACP; (**C**) LDH. All the data are presented as the means ± SDs (*n* = 6); * *p* < 0.01 and ** *p* < 0.01 compared to the model control group; # *p* < 0.01 and ## *p* < 0.01 compared to the normal control group. The grouping and abbreviations used are consistent with the descriptions provided earlier.

Compared to those in the NC group, those in the Cd group exhibited significantly greater ACP activity (*p* < 0.01; Figure 7B) and LDH activity (*p* < 0.05; Figure 7C) in testicular tissue. In contrast, ALP activity decreased significantly (*p* < 0.01; Figure 7A), disrupting glycolysis and affecting energy metabolism. These abnormal enzyme levels are associated with damage to spermatogenic cells. However, the ALP, ACP, and LDH levels were restored to their normal values in the VE and H-LAE groups, and they were significantly different from the levels recorded in the model group (*p* < 0.01).

### 2.8. Therapeutic LAE Alleviated Cd-Induced Oxidative Stress

**Figure 8:**
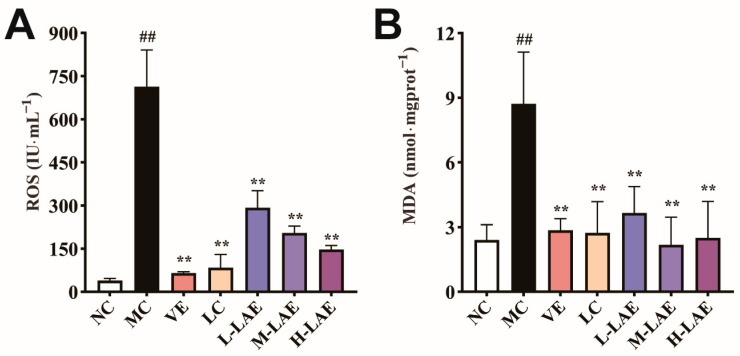
The serum ROS concentration and MDA concentration in testicular tissue homogenates are shown: (**A**) ROS; (**B**) MDA. All the data are presented as the means ± SDs (*n* = 6); ** *p* < 0.01 compared to the model control group; ## *p* < 0.01 compared to the normal control group. The grouping and abbreviations used are consistent with the descriptions provided earlier.

Compared with those in the NC group, cadmium-induced oxidative stress increased ROS levels in testicular tissue (*p* < 0.01; Figure 8A). The accumulated ROS damaged the cell membrane and interacted with unsaturated fatty acids, initiating lipid peroxidation and ultimately leading to the excessive production of MDA (*p* < 0.01; Figure 8B). The levels of ROS and MDA decreased significantly in all treatment groups.

**Figure 9:**
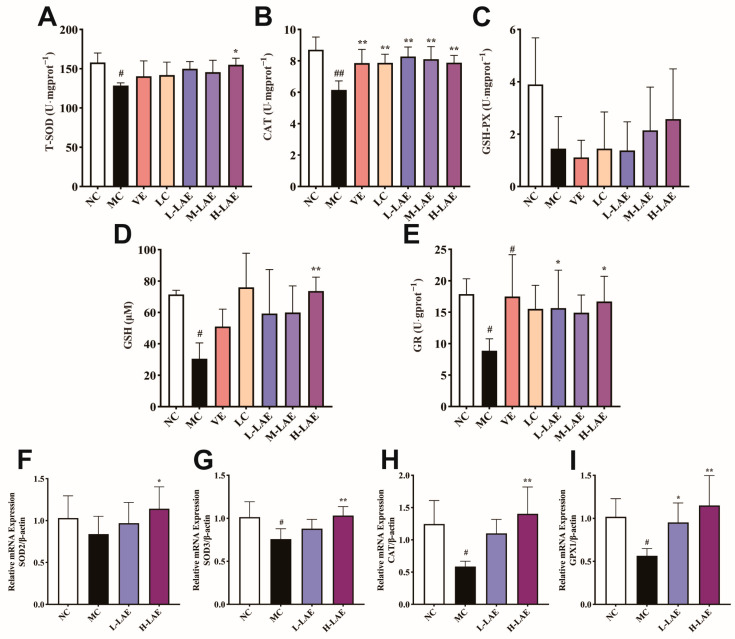
Changes in T-SOD, CAT, GSH-PX, GSH, and GR antioxidant enzyme activity. (**A**) T-SOD; (**B**) CAT; (**C**) GSH-PX; (**D**) GSH; (**E**) GR. Relative expression of the genes in the normal control group. (**F**) *SOD2* gene; (**G**) *SOD3* gene; (**H**) *CAT* gene; (**I**) *GPX1* gene. All the data are presented as the means ± SDs (*n* = 5–6); * *p* < 0.05 and ** *p* < 0.01 compared to the model control group; # *p* < 0.05 and ## *p* < 0.01 compared to the normal control group. The grouping and abbreviations used are consistent with the descriptions provided earlier.

In the MC group, the activity of antioxidant enzymes such as T-SOD, CAT, and GR decreased significantly, but the activity of GSH-PX did not significantly change (Figure 9A–C,E). The mRNA expression levels of the *SOD3*, *CAT*, and *GPX1* genes were also significantly downregulated in the MC group relative to their corresponding levels in the NC group (*p* < 0.05). However, no significant changes in the *SOD2* gene were found (Figure 9F). The H-LAE group exhibited significantly greater T-SOD and glutathione reductase (GR) activities (*p* < 0.05) and significantly greater *SOD3* and *GPX1* mRNA expression levels than the MC group (*p* < 0.01; Figure 9G,I). A decrease in the level of GR led to a reduction in the capacity to catalyze the conversion of oxidized glutathione (GSSH) to glutathione (GSH), resulting in a significant decrease in the GSH levels in testicular tissue (*p* < 0.05). However, the GSH levels increased in the H-LAE group (Figure 9D). In all the LAE treatment groups, CAT activity significantly increased, and the *CAT* gene expression increased (*p* < 0.01; Figure 9H).

### 2.9. Effects of the Keap1/Nrf2 Signaling Pathway on Gene Expression

**Figure 10:**
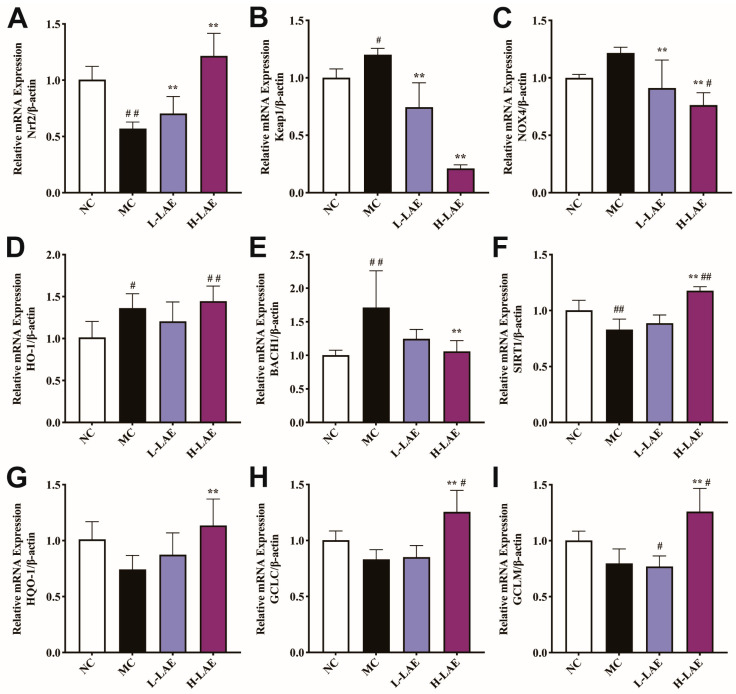
The relative mRNA expression levels in the normal control group. (**A**) *Nrf2*; (**B**) *Keap1*; (**C**) *NOX4*; (**D**) *HO-1*; (**E**) *BACH-1*; (**F**) *SIRT1*; (**G**) *HQO-1*; (**H**) *GCLC*; (**I**) *GCLM*. All the data are presented as the means ± SDs (*n* = 5–6); ** *p* < 0.01 compared to the model control group; # *p* < 0.05 and ## *p* < 0.01 compared to the normal control group. The grouping and abbreviations used are consistent with the descriptions provided earlier.

The *Keap1*/*Nrf2* axis is a key regulator of oxidative stress. To assess the mRNA expression of essential oxidative stress-related genes (Figure 10), we performed RT–qPCR. The mRNA levels of *Nrf2* and *SIRT1* were significantly lower in the Cd-exposed group than in the NC group (*p* < 0.01). In contrast, the mRNA levels of *Keap1* (*p* < 0.05), *HO-1* (*p* < 0.05), and *BACH1* (*p* < 0.01) were significantly greater in the Cd-exposed group than in the NC group. Compared to those in the MC group, the low-dose and high-dose LAE treatment groups exhibited significant upregulation of the *Nrf2* gene but significant downregulation of the *Keap1* and *NOX4* genes (*p* < 0.01). However, in the MC group, the changes in the expression of the *NOX4*, *NQO-1*, *GCLC*, and *GCLM* genes were not significant relative to those in the NC group. The expression of the *HO-1*, *GCLC*, and *GCLM* genes in the H-LAE group was significantly upregulated compared to that in the NC group.

### 2.10. Effects of the Keap1/Nrf2 Signaling Pathway on Protein Expression

**Figure 11:**
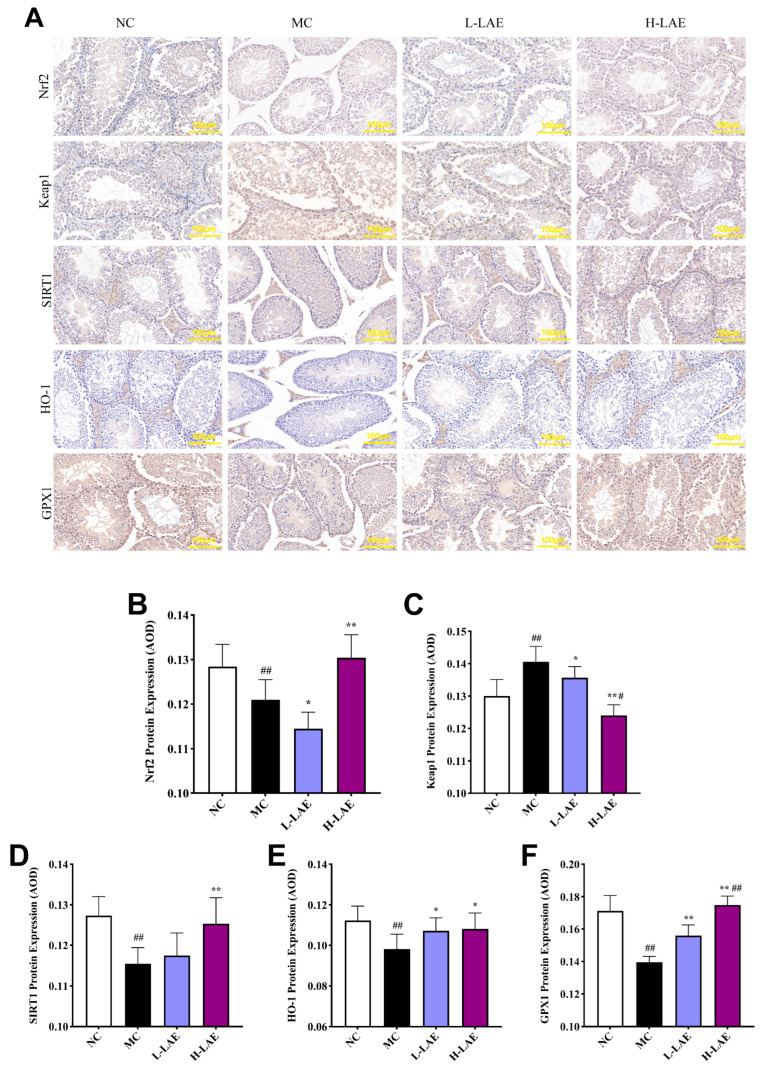
Immunohistochemical staining analysis of Nrf2, Keap1, SIRT1, HO-1, and GPX1 proteins. (**A**) Images illustrating the immunoreactivity of Nrf2, Keap1, SIRT1, HO-1, and GPX1; (**B**–**F**) the protein expression of Nrf2, Keap1, SIRT1, HO-1, and GPX1. All the data are presented as the means ± SDs (*n* = 3); * *p* < 0.05 and ** *p* < 0.01 compared to the model control group; # *p* < 0.05 and ## *p* < 0.01 compared to the normal control group. The grouping and abbreviations used are consistent with the descriptions provided earlier.

Immunohistochemistry (IHC) was conducted on cross-sections of paraffin-embedded testicular tissues; a brownish-yellow color indicated positive protein expression (Figure 11A). The average optical density (AOD) was calculated by assessing the ratio of the integrated optical density (IOD) value to the positive area, which allowed relative protein quantification (Figure 11B,C). In the MC group, a lower AOD of the Nrf2 protein was associated with weaker positive staining and a decrease in protein expression following Cd induction (*p* < 0.01). In contrast, the MC group exhibited greater AOD of the Keap1 protein, reflecting an increase in protein expression following Cd induction (*p* < 0.01). In the H-LAE group, an increase in the positive response to the SIRT1 and Nrf2 proteins was observed, with significantly greater protein expression (*p* < 0.01). Conversely, a significantly lower percentage of cells with a positive response to the Keap1 protein and significantly lower protein expression were noted (*p* < 0.01). Similarly, the L-LAE group presented significantly greater Nrf2 expression and significantly lower Keap1 protein expression (*p* < 0.05). The role of SIRT1 in deacetylating and activating the nuclear transcription factor Nrf2 was evident, as supported by the increased stable expression of Nrf2. LAE also decreased Keap1 levels, thereby activating Nrf2 through a reduction in ubiquitination via the Keap1/Nrf2 pathway. This process promoted the downstream expression of HO-1 and GPX1, resulting in elevated levels of antioxidant enzymes. Activated Nrf2 significantly increased the activity of antioxidant enzymes, such as *T*-SOD, CAT, and GR, while inhibiting MDA and ROS production. These changes mitigated oxidative damage to testicular tissue.

## 3. Discussion

In our study, chronic exposure to Cd led to systemic chronic poisoning, as indicated by a significant decrease in body weight after 21 days. The testicles are the male’s primary reproductive organs and are responsible for sperm production and the synthesis and secretion of several male hormones and growth factors in male mammals. The male reproductive system is susceptible to the toxic effects of Cd. Our experimental findings suggested that Cd exposure during adolescence can affect the development of male external genitalia, significantly reducing anogenital distance. Additionally, Cd inflicts severe damage to the male internal reproductive glands, resulting in a decrease in testis and epididymis organ indices and a reduction in organ quality. Histopathological examination of the testicular tissues revealed that the Leydig cells were damaged, as characterized by cell layer thickening, protein exudation, and cell apoptosis. These changes increase the intertubular space, weaken connections, and disrupt the integrity of the seminiferous tubules. Cd also damaged the seminiferous epithelium, leading to abnormal dilation or contraction of seminiferous tubules and severe structural damage to the testes; these findings are similar to those reported by Geng Xiao et al. [47]. However, after administering LAE, the extent of damage was reduced, which indicated that LAE had a significant protective effect on the testes.

This protective effect is achieved through the following steps. First, the interaction between Cd and calcium plays a key role. Cd inhibits the Ca^2+^-ATPase pump, promoting calcium influx into cells and causing intracellular calcium imbalance [48]. This triggers mitochondrial damage, leading to the generation of high levels of ROS. Cd competitively binds to calcium-binding proteins, disrupting calcium-dependent and calmodulin-dependent physiological processes, interfering with cell signal transduction pathways, and impairing protein tyrosine phosphorylation [49]. ALP is a biomarker for testicular function. A decrease in ALP levels is directly related to atrophy of the seminiferous tubules, a decrease in the mature sperm count in the tubules, shedding of seminiferous epithelium, and morphological abnormalities of Sertoli cells [50]. In our study, the activity of the testicular marker enzyme ALP decreased, which matched the results reported in other studies [51]. Similarly, the activity of ACP increased, which also matched the results reported in other studies [52,53]. This disparity might be attributed to variations in animal strains, ages, and Cd exposure doses. The abnormal activity of these enzymes affects the normal function of reproductive cells, leading to a decrease in T levels and sperm motility. Our results also revealed a significant decrease in the density of sperm motility and a greater proportion of abnormal sperm. Exposure to Cd resulted in severe disruption of spermatogenesis, leading to a high SDI (>1.2), which indicated the presence of severe spermatogenic disorders. L-carnitine is a water-soluble amino acid derivative that occurs naturally in the body. It is known for its ability to promote energy metabolism, increase sperm count, and reduce sperm abnormalities. A study reported that the protective effect of a high dose of LAE on spermatogenesis is similar to that of L-carnitine [54]. LAE can restore enzyme activity to normal levels, protect damaged seminiferous epithelium, and maintain the function of Leydig cells. The relatively intact seminiferous epithelium and tight Sertoli cell connections found in the H&E-stained sections further supported these findings.

Second, Cd directly attacks Leydig and Sertoli cells, causing DNA damage and cell apoptosis [55,56]. These cells synthesize and secrete male hormones. Cd also interferes with the HPGA, resulting in abnormal hormone levels. Our results showed that Cd significantly decreased T and INH-B levels and increased FSH levels. This enhanced negative feedback regulation, as INH-B levels negatively correlate with FSH levels. Our findings indicated that testosterone plays a key role in the development of the male reproductive system. Low levels of sex hormones can lead to reproductive cell dysfunction, apoptosis, and impairment of reproductive system development. LAE intervention modulated hormone levels and significantly increased T and INH-B levels compared to their respective levels in the model group; the T and INH-B levels were greater than their corresponding levels recorded in the normal control group. These results reflected the potential of LAE to enhance sperm production.

Cadmium (Cd) may interact with hydroxyl, thiol, and amino groups in proteins to form Cd-protein complexes, which disrupt the structure of enzymes and inhibit enzyme activity [57]. Cd can also replace zinc and manganese, reducing T-SOD activity [58]. Additionally, Cd can displace iron ions, decreasing CAT enzyme activity. Cd induces abnormal gene expression, inhibits DNA damage repair, and reduces the expression of the *SOD3*, *CAT*, and *GPX1* genes. A decrease in antioxidant enzyme activity leads to an excessive accumulation of superoxide radicals, which are converted to H_2_O_2_ as T-SOD activity decreases. A decrease in peroxidase activity leads to the accumulation of hydrogen peroxide, which in turn decreases GSH levels. GSH is a crucial non-enzymatic antioxidant that participates in redox reactions. Its thiol groups can bind to Cd, clearing heavy metals and protecting thiol enzymes. A reduction in GR activity further decreases the conversion of GSSH to reduced GSH, leading to a further decrease in GSH levels. This, in turn, leads to the production and accumulation of ROS and the lipid peroxidation product MDA, ultimately resulting in oxidative damage.

We used anthocyanin because it is an effective antioxidant. The antioxidant capacity of anthocyanins is linked to the number and position of hydroxyl substituents on their phenolic nucleus, conjugated groups, and glycosylation levels [59]. Anthocyanins can reduce the gene and protein expression of Keap1 and thus decrease its inhibitory effect on Nrf2. These changes can significantly upregulate the expression of the *Nrf2* gene, facilitating its nuclear translocation and activating the downstream genes *SOD3*, *CAT*, *HO-1*, *HQO1*, *GCLC*, and *GCLM*. Upregulating the expression of these genes enhances the activities of antioxidant enzymes such as T-SOD, CAT, and GR, promoting the clearance of ROS. Anthocyanins also increase GSH levels, directly scavenging hydrogen peroxide, singlet oxygen, and superoxide species in active oxygen clusters, thus reducing oxidative stress. Moreover, anthocyanins possess membrane-stabilizing abilities, protecting cell membranes from lipid peroxidation [60]. Anthocyanins can upregulate the expression of the SIRT1 gene, which is a crucial factor involved in cell aging and metabolism [61]. It can boost the activity of the Nrf2/Keap1/ARE pathway, help Nrf2 enter the cell nucleus, and enhance its transcriptional function. Thus, SIRT1 can protect Leydig cells, preventing a decrease in T production due to oxidative stress and restoring their ability to produce steroids. Additionally, a high level of the SIRT1 protein occurs in vascular endothelial cells, where it plays a key role in reducing oxidative stress and inflammation in blood vessels [62]. An in vivo monitoring system for vascular microcirculation (Micro-VCC, Optoprobe, Pontypridd, UK) was used to monitor changes in testicular microvessels. We also found that LAE can ameliorate testicular vascular constriction caused by Cd exposure, and this improvement might be linked to an increase in the expression of the SIRT1 protein. Our other results showed that anthocyanins can chelate heavy metal ions; their in vitro chelation potential for Cd^2+^ was similar to that of EDTA. They can effectively reduce Cd absorption in the intestine and alter Cd distribution in the body. We aim to investigate these mechanisms in the future.

## 4. Materials and Methods

### 4.1. Preparation of LAE

The dried fruits were identified as *Lycium ruthenicum* Murray and obtained from Panda Health Management Co., Ltd. (Beijing, China). The fruits were cultivated in Qinghai Province, China.

Following our previously established procedures, whole fruits were crushed, soaked in a 20% ethanol solution (EtOH: H_2_O = 2:8, *v*/*v*) with a material-to-liquid ratio of 1:6 for 30 min, stirred for 120 min, extracted twice with ultrasonication (500 W, 40 kHz), and filtered through a 200 mesh sieve (74 µm). The filtrates were pooled and concentrated under a reduced pressure of 0.09 MPa to 10% of the original volume at 50 °C. Further enrichment and purification were performed using macroporous adsorption resin [63]. The resin was washed twice the pure water bed volume (BV) to elute the protein. Other impurities were eluted using a 15% ethanol solution (EtOH:H_2_O = 3:17, *v*/*v*) twice with BV. The sample was collected using a 60% ethanol solution (EtOH:H_2_O = 6:4, *v*/*v*) with four times BV. Finally, the sample was concentrated. Vacuum freeze-drying was performed at −60 °C to obtain anthocyanin extract powder [64]. The extract was stored in a dry, cool, and dark environment [65].

### 4.2. Materials and Chemicals

Cadmium chloride anhydrous (CdCl_2_) was purchased from Strem Chemicals, Inc. (purity > 99.995%; Newburyport, MA, USA). All standard materials (purity > 99%, IsoReag) were purchased from MetwareBio Co., Ltd. (Wuhan, China). Methanol and formic acid were of chromatographic grade. Vitamin E was purchased from Beijing Solarbio Science & Technology Co., Ltd. (Beijing, China). L-carnitine oral solution was purchased from Northeast Pharmaceutical Group Shenyang No. 1 Pharmaceutical Co., Ltd. (Shenyang, China). T-SOD, CAT, GSH-PX, MDA, GSH, GR, and quick sperm stain test kits were purchased from Nanjing Jiancheng Bioengineering Institute (Nanjing, China). Enzyme-linked immunosorbent assay (ELISA) test kits for total testosterone (T), estradiol (E2), luteinizing hormone (LH), follicle-stimulating hormone (FSH), progesterone (P), and inhibin B (INH-B) were purchased from Shanghai Enzyme-linked Biotechnology Co., Ltd. (Shanghai, China). The ROS levels were measured using mouse ROS ELISA kits (Lot. ml024136, Shanghai Enzyme-linked Biotechnology Co., Ltd., China). Acid phosphatase (ACP), lactate dehydrogenase (LDH), alkaline phosphatase (ALP/AKP), and total protein (TP) test kits were purchased from Biosino Bio-Technology and Science Inc. (Beijing, China). The primers for *Nrf2*, *Keap1*, *HO-1*, *NQO-1*, *SOD2*, *SOD3*, *CAT*, *GCLC*, *GCLM*, *BACH1*, *SIRT1*, *NOX4*, *GPX1*, and *β-actin* were synthesized and purified by GenScript Biotech Corp. (Nanjing, China). Total animal RNA easy-fast extraction kits were purchased from Tiangen Biotech Co., Ltd. (Beijing, China). Prime Script RT Master Mix and TB Green Premix Ex Taq (SYBR) were purchased from Takara Bio Inc. (Kyoto, Japan). Antibodies were purchased from Beijing Biosynthesis Biotechnology Co., Ltd. (Beijing, China). All the other chemicals and reagents used in this study were of analytical grade and were purchased from local suppliers.

### 4.3. Determination of Total Anthocyanin Content

The total anthocyanin content (TAC) in LAE was determined using a modified pH differential method and expressed as petunidin (Pt) [66,67]. LAE (0.1 g) was weighed and added to 50 mL of the extraction solution (HCl: 80% EtOH = 3:97, *v*/*v*). The mixture underwent 30 min of ultrasonic extraction, followed by centrifugation at 8000 rpm for 3 min. The supernatant was collected, diluted five times, and used as the sample solution. The two sample solutions were each diluted five times; one solution was diluted with buffer at pH 1.0 (0.025 M potassium chloride), and the other was diluted with buffer at pH 4.5 (0.4 M sodium acetate). The absorbance was recorded at 532 nm and 700 nm, and each analysis was conducted in triplicate. The TAC was expressed as petunidin (Pt) equivalents (Pt g/100 g) and calculated using Equation (1), as shown below:

(1) $$TAC\;(Ptg/100g)=\frac{∆A\times MW\times DF\times V}{\varepsilon \times l\times m\times 10}$$

Here, ∆A indicates the difference between A_532_ nm and A_700_ nm at pH 1.0 and A_532_ nm and A_700_ nm at pH 4.5; MW indicates the molecular weight (912.7 g/mol for the average molar mass of Pt); DF indicates the dilution factor; V indicates the extraction volume (mL); ε indicates the molar extinction coefficient (29,591 for Pt, L·mol^−1^·cm^−1^); l indicates the thickness of the colorimetric dish (1 cm); m indicates the sample weight (g); and 10 represents the factor for conversion from g to 100 g [68].

### 4.4. Characterization of Anthocyanins

The LAE was analyzed qualitatively and quantitatively via ultra-performance liquid chromatography–tandem mass spectrometry (UHPLC-MS/MS). The ExionLC™ AD system consisted of a high-speed pump, a multiple autosampler, and a photodiode array detector. The instrument was connected to an IonDrive Turbo V electrospray ionization (ESI) tandem QTRAP 6500+ mass spectrometry system (AB Sciex Pte. Ltd., Framingham, MA, USA). To prepare the samples, 50 mg of LAE was weighed and dissolved in a 0.5 mL solution containing 50% methanol in water with 0.1% HCl. The solution was vortexed for 5 min, sonicated for 5 min, and subsequently centrifuged at 12,000 rpm for 3 min at 4 °C. After the supernatant was carefully decanted, this procedure was repeated. The two supernatant fractions were pooled and filtered through a 0.22 µm microporous filter membrane. The sample was separated using a reversed-phase ACQUITY UPLC BEH C18 column (2.1 mm × 100 mm, 1.7 µm; Waters Inc., Milford, MA, USA). Mobile phase A (water) and phase B (methanol) were acidified with 0.1% formic acid. The gradient elution program used was as follows: 0–6 min, 5–50% B; 6–12 min, 50–95% B; 12–14 min, 95% B; and 14–16 min, 5% B. The flow rate was 0.35 mL/min. The column temperature was 40 °C, and the optimum absorption wavelength was 525 nm. The samples (2 µL) were injected into the system, and mass analyses were performed in positive ionization mode. The ESI-MS parameters were set as follows: ESI ion source, temperature = 550 °C, ion spray voltage = 5500 V (+), curtain gas = 35 psi, and a high collision-induced ionization parameter. The full MS scan range was set from 100 to 1200 m/z. A database of standards (MetwareBio Co., Ltd., Wuhan, China) was used to qualitatively analyze the mass spectrometry data. The total ion current (TIC) was recorded, and secondary spectra of the matching components were determined. The analysis utilized a triple quadrupole mass spectrometer’s multiple reaction monitoring (MRM) modes. This involved capturing the signal intensities of chromatographic peaks corresponding to standards of different concentrations. A standard curve was constructed, plotting concentration on the *X*-axis and the peak area on the *Y*-axis, facilitating the analysis of various components [69,70].

### 4.5. Animals and Experimental Design

ICR male mice (6–8 weeks old, 18–22 g) were obtained from Beijing HFK Bioscience Co., Ltd. (Beijing, China). The mice were housed in a specific pathogen-free (SPF) barrier system under controlled conditions; the room temperature was maintained at 20–26 °C, with the humidity was 40–70%, and the light/dark cycle was 12-h/12-h. All mice were provided a standard diet ad libitum. The animal experimental protocols and care complied with ethical guidelines and were approved by the animal ethics committee of the Institute of Medicinal Plant Development, which is affiliated with the Chinese Academy of Medical Sciences (approval number: SLXD-20230323014). All the mice were acclimated for one week before the experiment.

Male mice (*n* = 70) were randomly selected and equally distributed into seven groups, with 10 mice in each group. The experiment lasted for 28 days. The groups were established as follows: (1) Mice in the normal control (NC) group received 0.9% NaCl daily via gavage. (2) Mice in the model control (MC) group were administered CdCl_2_ (5 mg/kg bwt) dissolved in distilled water via gavage. (3) Mice in the vitamin E positive control (VE) group received daily gavage of CdCl_2_ (5 mg/kg) with vitamin E (100 mg/kg bwt) in corn oil. (4) Mice in the L-carnitine positive control (LC) group were gavaged daily with CdCl_2_ (5 mg/kg) and L-carnitine oral solution (500 mg/kg bwt). (5) Mice in the low-dose treatment group (L-LAE) received a daily gavage of CdCl_2_ (5 mg/kg) and LAE (50 mg/kg bwt) dissolved in distilled water. (6) Mice in the medium-dose treatment group (M-LAE) received a daily gavage of CdCl_2_ (5 mg/kg) and LAE (250 mg/kg bwt) dissolved in distilled water. (7) Mice in the high-dose treatment group (H-LAE) received a daily gavage of CdCl_2_ (5 mg/kg) and LAE (500 mg/kg bwt) dissolved in distilled water. The body weights of the mice were monitored every four days. The selected doses were determined based on preliminary research and published studies [71,72,73,74].

### 4.6. Sample Preparation

After the experiment was concluded (28 days), the mice were starved for 12 h. Then, they were weighed and anesthetized with 1.25% 2,2,2-tribromoethanol (0.2 mL/10 g) before being humanely euthanized by cervical dislocation. The anogenital distance index, which indicates the distance from the anus to the penis, was measured using a Vernier caliper. The testes and epididymides were excised, cleared of excess adipose tissues, and weighed. The relative weights of the organs were calculated by dividing the absolute organ weight by the final body weight, and the results are expressed as a percentage [75]. The left testicle was placed in 4% paraformaldehyde fixative for histological examination. The right testicle was partially removed and used to create testicular homogenates. These organs were rapidly frozen in liquid nitrogen and stored at –80 °C. Whole-blood samples were collected, allowed to clot for 60 min, and subsequently centrifuged at 3500 rpm for 15 min to separate the serum for subsequent analyses.

### 4.7. Analysis of Sperm Parameters

Initially, the cauda epididymis was collected in 3.0 mL of 0.9% NaCl, dissected into smaller pieces to facilitate the release of sperm, and then incubated at 37 °C for 30 min [76]. Before assessing the parameters of the sperm cells, the sperm suspension was gently agitated. A 20 µL sperm suspension was placed on a sperm counting board (with a depth of 10 µm) and subjected to comprehensive sperm analysis using a computer-aided semen analysis system (CASA, JFX-A/B, Huazhong Medical Products Co., Ltd., Shijiazhuang, China), which included assessments of sperm motility, density, and the ‘a + b’ sperm ratio. The sperm density was calculated as the total sperm count divided by the volume of the semen solution (10^6^/mL). Sperm motility represents the proportion of viable sperm relative to the total sperm count. Grade ‘a’ sperm cells include those with rapid progressive motion at a velocity of 25 µm/s at 37 °C, while Grade ‘b’ sperm cells include those with slower but still progressive motion at a velocity of 5–25 µm/s at 37 °C [77]. The sperm quality analysis adhered to the guidelines provided in the sixth edition of the WHO laboratory manual for examining and processing human semen [78].

The sperm suspension was centrifuged at 1000 rpm for 5 min, the supernatant was discarded, and the sperm pellet was resuspended in a small quantity of M199 medium. A sperm suspension (20 µL) was deposited on a clean glass slide, spread evenly, and allowed to air-dry overnight. Following the guidelines provided with the Quick Sperm Stain Kit, we made permanent sperm smears using the modified Papanicolaou staining method. Photographs of all the slides were taken in five randomly selected microscopic fields at 400× magnification, with a minimum of 40 sperm cells per field. We recorded morphologically abnormal sperm cells as previously described. The evaluation of abnormal sperm morphology followed the WHO guidelines and included the teratozoospermia index (TZI), calculated as the total number of defects divided by the number of abnormal sperm, and the sperm deformity index (SDI), calculated as the total number of defects divided by the total number of sperm [79].

### 4.8. Determination of the Concentration of Hormones

The serum samples were diluted and processed following the protocol provided with the ELISA kit. A standard curve was constructed by plotting the standard concentrations of sex hormones on the *X*-axis and their corresponding optical density (OD) values on the *Y*-axis. The absorbance was measured at 450 nm using a microplate reader (Tecan Trading AG, Infinite M1000, Männedorf, Switzerland), and the standard curve was used to determine sample concentrations. T, LH, FSH, E2, P, and INH-B concentrations in the serum were quantified.

### 4.9. Determination of Oxidative/Antioxidant Parameters

The testicular tissue samples were accurately weighed (0.1 g) and homogenized in 1 mL of precooled 0.9% NaCl. The homogenate was then centrifuged at 3000 rpm for 15 min at 4 °C to collect the tissue supernatant. The oxidation/antioxidation status was assessed using commercial kits to determine the activities of antioxidant enzymes, including T-SOD, CAT, GSH-PX, and GR, as well as the level of the non-enzymatic antioxidant GSH and the lipid peroxidation product MDA.

### 4.10. Determination of Testicular Marker Enzymes

A fully automatic biochemical analyzer (Beckman Coulter, Inc., West Sacramento, CA, USA) was used to measure the ACP, LDH, and ALP levels and the total protein concentration in the testicular tissue homogenates.

### 4.11. Analysis of ROS

The level of ROS in the serum was measured using a commercially available kit, as described in Section 4.8.

### 4.12. Histopathological Observation

The fixed testicles were dehydrated in graded ethanol, cleaned with xylene, and embedded in a paraffin block. Then, the sections (3–5 µm thick) were stained with hematoxylin and eosin (H&E), sealed with a mixture of neutral gum and xylene, and examined using a Nikon Eclipse Ci-L optical microscope (Nikon Co., Tokyo, Japan). The sections were assessed for any alterations in their morphological structure, number or structure of Sertoli and Leydig cells, wall thickness, diameter of the lumen of seminiferous tubules, or spermatogenesis. Johnsen’s mean testicular biopsy score (JMTBS) was determined for at least 50 seminiferous tubules to evaluate the extent of testicular damage. The scoring criteria for the JMTBS are presented in Table 2 [80].

### 4.13. Gene Expression

Total RNA was extracted from 15 mg of the testicular tissue sample using a silicon matrix spin column RNA easy fast tissue kit. The purity and concentration of the total RNA were measured using a Nanodrop 2000 ultra-micro-volume spectrophotometer (Thermo Fisher Scientific Inc., Waltham, MA, USA). The OD 260/280 nm ratio was between 1.8 and 2.0, which indicated low protein contamination and high RNA purity. The integrity of the RNA was determined by electrophoresis on a 2% agarose gel. Total RNA was reverse-transcribed into cDNA, which was used as a template for further amplification. An ABI Quantstudio 6 Flex Real-Time PCR System (Applied Biosystems, Inc., Carlsbad, CA, USA) and TB Green Premix Ex Taq PCR Kits were used to perform RT–qPCR. The primers used for *Nrf2*, *Keap1*, *HO-1*, *NQO-1*, *SOD2*, *SOD3*, *CAT*, *GCLC*, *GCLM*, *BACH1*, *SIRT1*, *NOX4*, and *GPX1* were designed by using the National Center for Biotechnology Information (NCBI) Primer Blast Database (http://www.ncbi.nlm.nih.gov/tools/primer-blast on 6 April 2023), as presented in Table 3, and *β-actin* was used as a housekeeping gene. For real-time quantitative polymerase chain reaction (RT–qPCR), 12.5 µL of total reaction mixture was used, which included 6.25 µL of TB Green Premix Ex Taq II (2×, Conc.), 0.5 µL of forward and reverse primers (10 µM), 4 µL of cDNA template, 0.25 µL of ROX Reference Dye II (50×, Conc.), and 1.5 µL of RNase-free water. The RT–qPCR procedure included preincubation at 95 °C for 30 s; 40 amplification cycles of denaturation at 95 °C for 10 s, annealing at 60 °C for 10 s, and extension at 72 °C for 10 s; and a final melting at 95 °C for 30 s. Amplification and melting curves were generated. The cycle threshold (Ct) value was used to calculate the relative gene expression using the 2^−∆∆CT^ method. The RT–qPCR amplification plots and melt curve plots for the specified genes are illustrated in Figures S1 and S2, respectively.

### 4.14. Immunohistochemistry

Immunohistochemistry was performed using paraffin sections of the testicles to evaluate the protein levels of Nrf2, Keap1, SIRT1, HO-1, and GPX1 (Servicebio, Wuhan, China). Before antigen retrieval, the paraffin sections were dewaxed in xylene, hydrated in an ethanol gradient, and placed in a citric acid antigen retrieval buffer (pH 6.0). The sections were then incubated in a 3% hydrogen peroxide solution for 25 min to block endogenous peroxidase activity, followed by three washes with phosphate-buffered saline (PBS at pH 6.0). Next, 3% bovine serum albumin (BSA) was added to the slides, which were incubated for 30 min to prevent the binding of endogenous nonspecific protein antigens. After removing the blocking solution, the sections were incubated with primary antibodies at 4 °C overnight and washed with PBS. Then, the appropriate horseradish peroxidase (HRP) secondary antibodies were added to the sections, which were incubated at room temperature for 50 min. Finally, the sections were stained using a DAB chromogenic solution, counterstained with hematoxylin for nuclear contrast, dehydrated, and sealed for analysis. The sections were observed using a Nikon E100 optical microscope (Nikon Co., Tokyo, Japan) and analyzed using ImageJ 1.53t (Rawak Software Inc., Stuttgart, Germany).

### 4.15. Statistical Analysis

Statistical analysis and data visualization were conducted using GraphPad Prism 9 (GraphPad Software, Inc., San Diego, CA, USA). The differences between the experimental groups were determined by one-way analysis of variance (ANOVA) and Tukey’s multiple comparison test. All the data are presented as the mean ± standard deviation (SD). All differences were considered to be statistically significant at *p* < 0.05 (* *p* < 0.05 and ** *p* < 0.01).

## 5. Conclusions

The anthocyanins found in *Lycium ruthenicum* Murray can effectively reduce Cd-induced oxidative stress in testicles. These compounds can effectively alleviate testicular pathological damage, maintain germ cell homeostasis, improve sperm quality, restore testosterone levels, and mitigate the reproductive toxicity associated with Cd exposure by activating the Keap1/Nrf2 antioxidant pathway.

## Figures and Tables

**Table 1:** Anthocyanins in LAE.

Identification	tR	Formula	Ionization Model	Molecular	Content (μg/g)
Procyanidin B1	4.21	C_30_H_26_O_12_	[M+H]^+^	578.14	2.21
Cyanidin-3,5-O-diglucoside	5.89	C_27_H_31_O_16_^+^	[M]^+^	611.16	45.13
Procyanidin B2	5.96	C_30_H_26_O_12_	[M+H]^+^	578.14	5.05
Delphinidin-3-O-sophoroside	6.46	C_27_H_31_O_17_^+^	[M]^+^	627.16	16.40
Delphinidin-3-O-galactoside	6.50	C_21_H_21_O_12_^+^	[M]^+^	465.10	80.22
Cyanidin-3-gentiobioside	6.90	C_27_H_31_O_16_^+^	[M+H]^+^	611.16	6.76
Delphinidin-3-O-glucoside	6.95	C_21_H_21_O_12_^+^	[M]^+^	465.10	51.98
Cyanidin-3-O-arabinosidase-glucoside	7.15	C_26_H_29_O_15_^+^	[M+H]^+^	581.15	1.90
Cyanidin-3-O-galactoside	7.43	C_21_H_21_O_11_^+^	[M]^+^	449.11	169.64
Delphinidin-3-O-rutinoside-5-O-glucoside	7.48	C_33_H_41_O_21_^+^	[M]^+^	773.21	28.77
Delphinidin-3-O-rutinoside	7.57	C_27_H_31_O_16_^+^	[M]^+^	611.16	23.11
Cyanidin-3-O-sambubioside	8.11	C_26_H_29_O_15_^+^	[M]^+^	581.15	19.49
Pelargonidin-3-O-galactoside	8.32	C_21_H_21_O_10_^+^	[M]^+^	433.11	3.55
Petunidin-3-O-sophoroside	8.60	C_28_H_33_O_17_^+^	[M]^+^	641.17	5.77
Cyanidin-3-O-rutinoside	8.62	C_27_H_31_O_15_^+^	[M]^+^	595.17	181.40
Petunidin-3-O-galactoside	8.63	C_22_H_23_O_12_^+^	[M]^+^	479.12	23.00
Petunidin-3-O-glucoside	8.64	C_22_H_23_O_12_^+^	[M]^+^	479.12	12.14
Peonidin-3-O-galactoside	9.02	C_22_H_23_O_11_^+^	[M]^+^	463.12	12.17
Petunidin-3-O-sophoroside-glucoside-5-O-sambubioside	9.06	C_45_H_61_O_31_^+^	[M+H]^+^	1097.32	3.38
Cyanidin-3-O-(6″-O-acetyl-2″-O-xylosyl) glucoside	9.53	C_28_H_31_O_16_^+^	[M+H]^+^	623.16	13.59
Malvidin-3-O-galactoside	9.54	C_23_H_25_O_12_^+^	[M]^+^	493.13	58.48
Cyanidin-3-O-xyloside	10.24	C_20_H_19_O_10_^+^	[M]^+^	419.10	57.73
Malvidin-3-O-rutinoside	10.32	C_29_H_35_O_16_^+^	[M]^+^	639.19	130.37
Delphinidin-3-O-(6″-O-coumaroyl) rhamnoside-5-O-glucoside	10.42	C_36_H_37_O_18_^+^	[M+H]^+^	757.20	9.86
Cyanidin-3-O-(6-O-malonyl-beta-D-glucoside)	10.77	C_24_H_23_O_14_^+^	[M]^+^	535.11	11.43
Petunidin-3-O-rutinoside-5-O-rhamnoside	10.97	C_34_H_43_O_20_^+^	[M+H]^+^	771.23	4.11
Quercetin-3-O-glucoside	11.71	C_21_H_20_O_12_	[M+H]^+^	464.10	155.23
Cyanidin-3-O-(6″-O-caffeoyl) rhamnoside	12.65	C_30_H_27_O_13_^+^	[M+H]^+^	595.15	8.65
Petunidin-3-O-(6-O-p-coumaroyl)-glucoside	12.74	C_31_H_29_O_14_^+^	[M]^+^	625.16	5.00
Naringenin	13.17	C_15_H_12_O_5_	[M+H]^+^	272.07	11.55

**Table 2:** The standard Johnsen’s mean testicular biopsy score (JMTBS).

Score	Standard
10	Complete spermatogenesis and perfect tubule
9	Many spermatozoa present, but germinal epithelium disorganized with marked sloughing or obliteration of lumen
8	Only a few (<5–10) late spermatids
7	No late spermatids, but many early spermatids
6	No spermatozoa and only few spermatids (<5–10) present
5	No spermatozoa, no spermatids, but several or many spermatocytes present
4	Only few spermatocytes (<5) and no spermatids or spermatozoa present
3	Spermatogonia are the only germ cells present
2	No germ cells but Sertoli cells are present
1	No cells in tubular section

**Table 3:** The sequences of the primers used in RT–qPCR.

Gene	Species	Forward (5′-3′)	Reverse (3′-5′)
*β-actin*	Mouse	TATAAAACCCGGCGGCGCA	CATCCATGGCGAACTGGTGG
*Nrf2*	Mouse	ACCTCTGCTGCAAGTAGCCT	TGGGCAACCATCACTCTGCT
*Keap1*	Mouse	ACAGCAGCGTGGAGAGATATG	GTTAAGCCGGTTAGTCCCGT
*HO-1*	Mouse	GCTAGCCTGGTGCAAGATACT	AAGCTGAGAGTGAGGACCCA
*NQO-1*	Mouse	AGGATGGGAGGTACTCGAATC	AGGCGTCCTTCCTTATATGCTA
*SOD2*	Mouse	TCGCTCTTCAGCCTGCACTG	AGCCTCCAGCAACTCTCCTTTG
*SOD3*	Mouse	TGAGAAGATAGGCGACACGC	TGGCTGATGGTTGTACCCTG
*CAT*	Mouse	CGCAATTCACACCTACACGC	GGGAGAATCCATCCAGCGTT
*GCLC*	Mouse	GGGGTGACGAGGTGGAGTA	GTTGGGGTTTGTCCTCTCCC
*GCLM*	Mouse	CAATGACCCGAAAGAACTGCTC	TTCCCCTGCTCTTCACGATG
*BACH1*	Mouse	AGAGGGAGTGAGTCACCTG	TCACTGTCATCGACCGGAG
*SIRT1*	Mouse	TCGGCTACCGAGGTCCATA	TAACAATCTGCCACAGCGTC
*NOX4*	Mouse	ACCAAATGTTGGGCGATTGTG	GATGAGGCTGCAGTTGAGGT
*GPX1*	Mouse	AGTCCACCGTGTATGCCTTCT	GAGACGCGACATTCTCAATGA

## Data Availability

Data are contained within the article.

## Supplementary Materials

The following supporting information can be downloaded at https://www.mdpi.com/article/10.3390/ph17030322/s1. Figure S1: Amplification plots; Figure S2: Melt curve plots.
