# Supplementary material for: Anthocyanins from Lycium ruthenicum Murray Mitigate Cadmium-Induced Oxidative Stress and Testicular Toxicity by Activating the Keap1/Nrf2 Signaling Pathway
Source: Pharmaceuticals (Basel). 2024 Mar 1;17(3):322. doi: 10.3390/ph17030322 (PMC10975946; doi:10.3390/ph17030322)
Supplement: Supplementary file 1 [file pharmaceuticals-17-00322-s001.zip › pharmaceuticals-2856435-Supplementary.pdf]

Supplementary Materials

Figure S1. The amplification plots of β-actin, Keap1, and Nrf2.

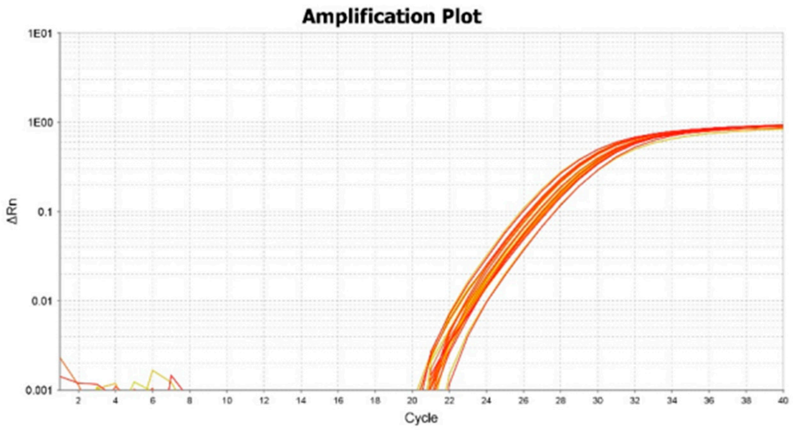

.

Amplification plot of β-actin

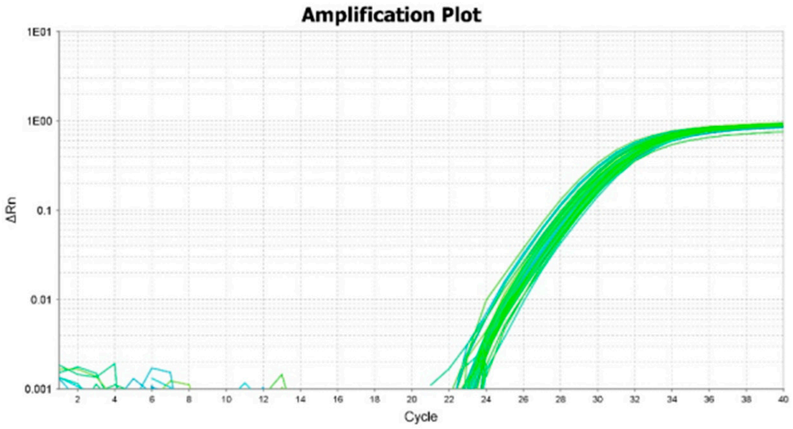

Amplification plot of Nrf2

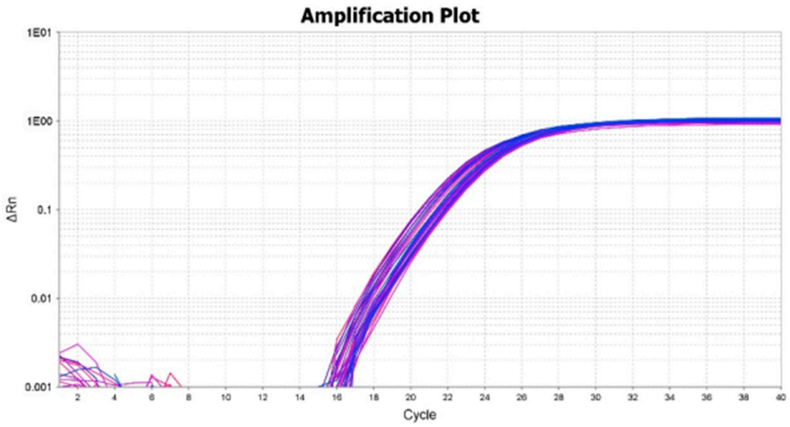

Amplification plot of Keap1

Figure S2. The melt curve plots of β-actin, Nrf2, Keap1, HO-1, SOD2, SOD3, CAT, BACH1, SIRT1, NOX4, GPX1.

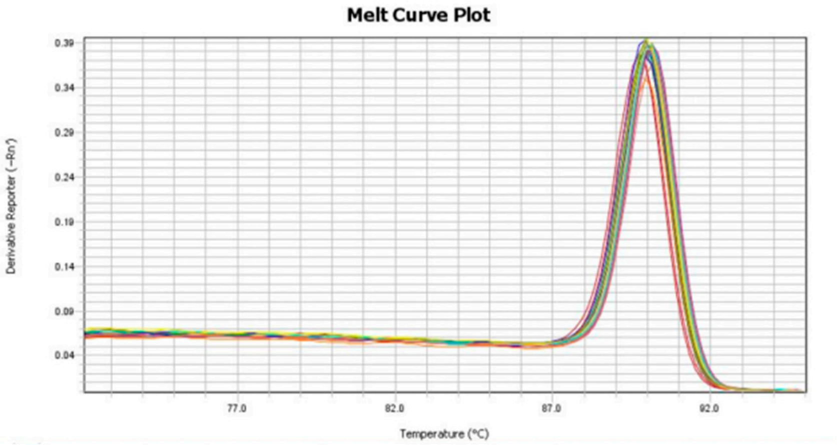

Melt curve plot of β-actin

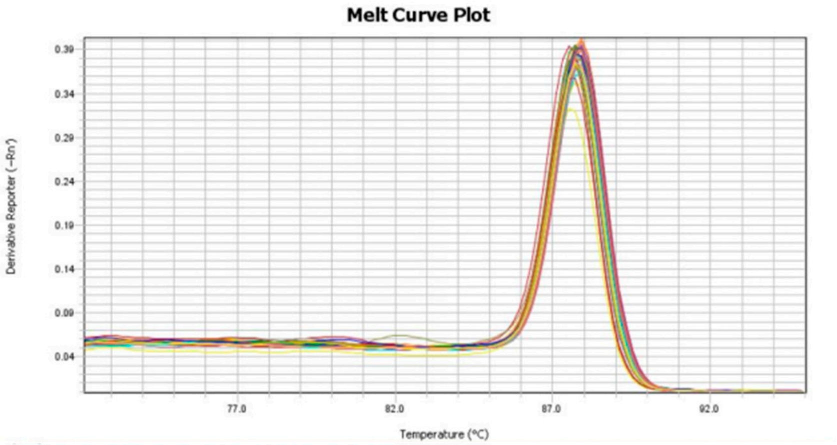

Melt curve plot of Nrf2

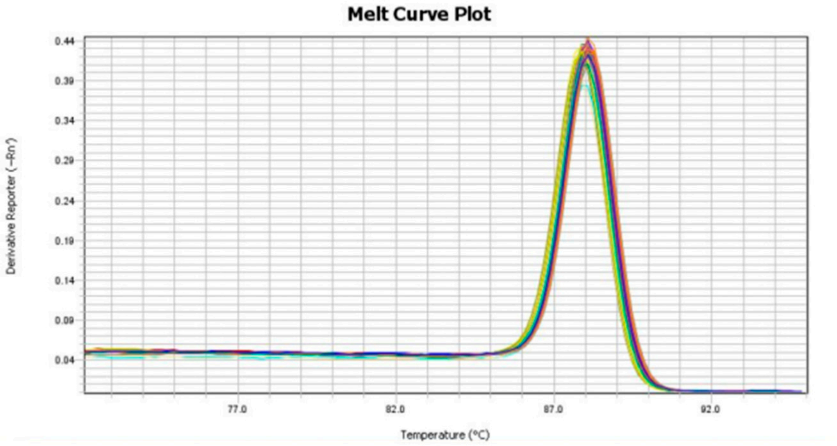

Melt curve plot of Keap1

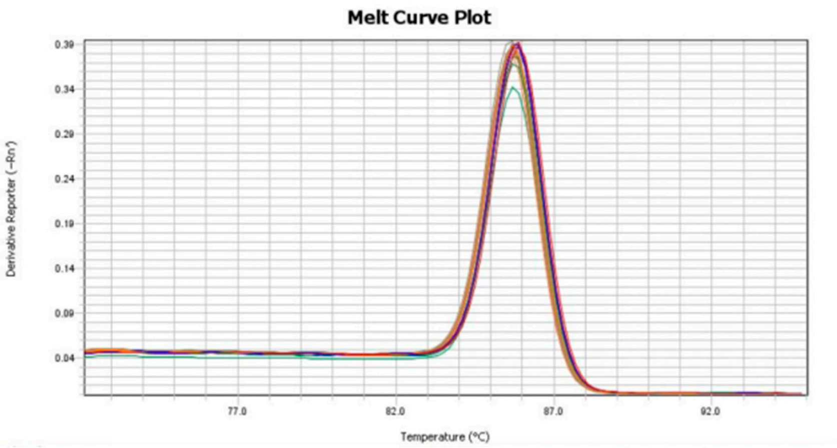

Melt curve plot of HO-1

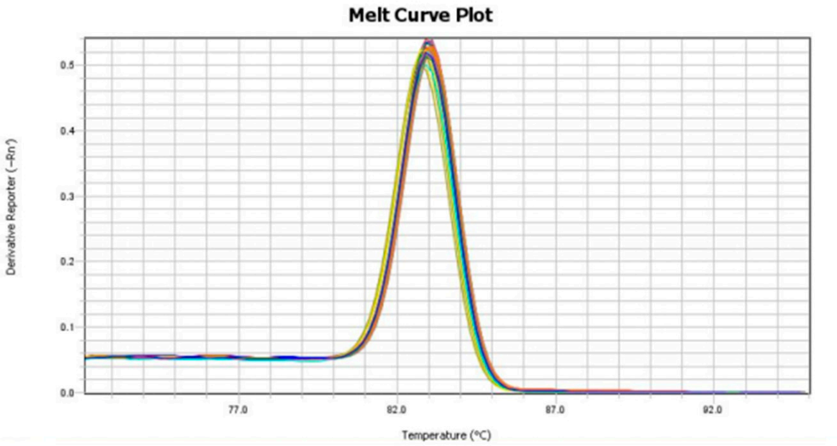

Melt curve plot of SOD2

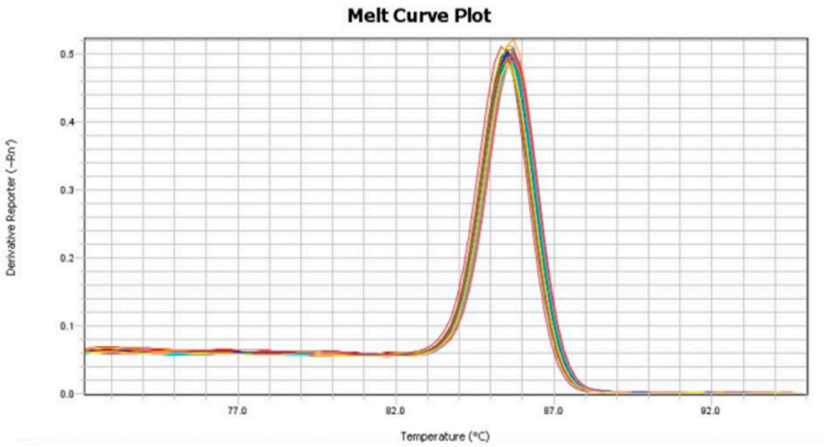

Melt curve plot of SOD3

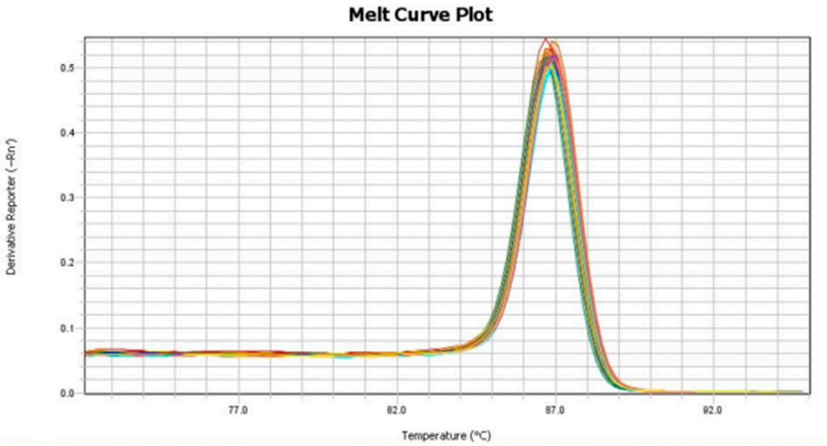

Melt curve plot of CAT

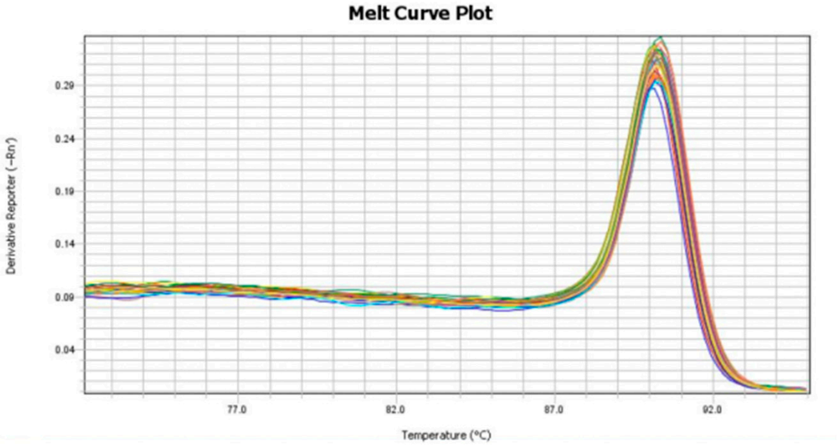

Melt curve plot of BACH1

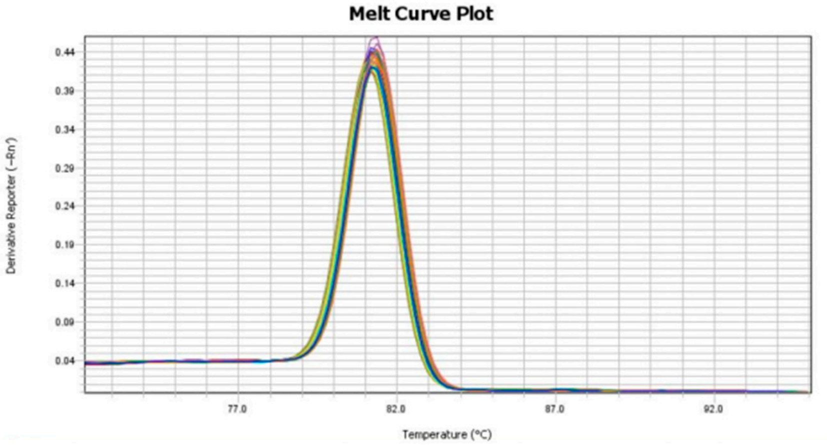

Melt curve plot of SIRT1

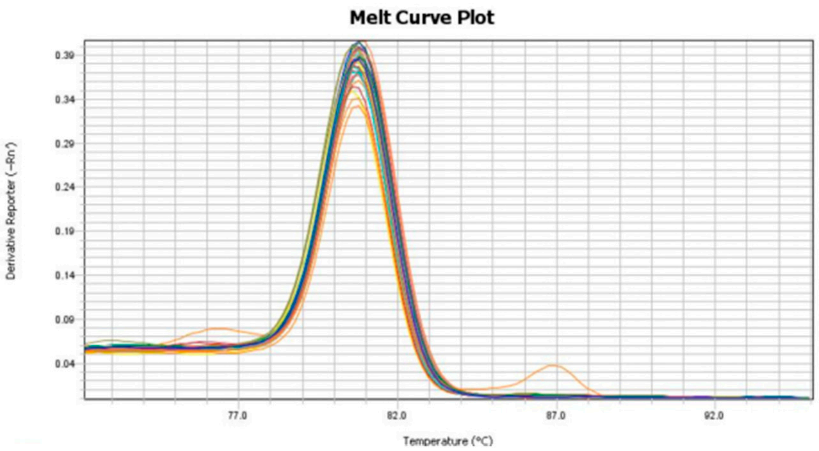

Melt curve plot of NOX4

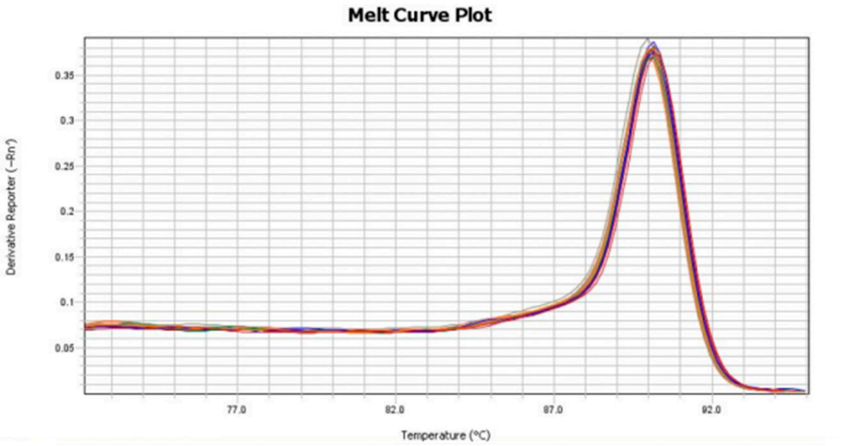

Melt curve plot of GPX1
